# Effects of tissue degradation by collagenase and elastase on the biaxial mechanics of porcine airways

**DOI:** 10.1186/s12931-023-02376-8

**Published:** 2023-04-08

**Authors:** Crystal A. Mariano, Samaneh Sattari, Gustavo O. Ramirez, Mona Eskandari

**Affiliations:** 1 Department of Mechanical Engineering, University of California at Riverside, Riverside, CA USA; 2 BREATHE Center, School of Medicine, University of California at Riverside, Riverside, CA USA; 3 Department of Bioengineering, University of California at Riverside, Riverside, CA USA

**Keywords:** Elastase, Collagenase, Biaxial testing, Airway tissue mechanics, Pulmonary biomechanics

## Abstract

**Background:**

Common respiratory illnesses, such as emphysema and chronic obstructive pulmonary disease, are characterized by connective tissue damage and remodeling. Two major fibers govern the mechanics of airway tissue: elastin enables stretch and permits airway recoil, while collagen prevents overextension with stiffer properties. Collagenase and elastase degradation treatments are common avenues for contrasting the role of collagen and elastin in healthy and diseased states; while previous lung studies of collagen and elastin have analyzed parenchymal strips in animal and human specimens, none have focused on the airways to date.

**Methods:**

Specimens were extracted from the proximal and distal airways, namely the trachea, large bronchi, and small bronchi to facilitate evaluations of material heterogeneity, and subjected to biaxial planar loading in the circumferential and axial directions to assess airway anisotropy. Next, samples were subjected to collagenase and elastase enzymatic treatment and tensile tests were repeated. Airway tissue mechanical properties pre- and post-treatment were comprehensively characterized via measures of initial and ultimate moduli, strain transitions, maximum stress, hysteresis, energy loss, and viscoelasticity to gain insights regarding the specialized role of individual connective tissue fibers and network interactions.

**Results:**

Enzymatic treatment demonstrated an increase in airway tissue compliance throughout loading and resulted in at least a 50% decrease in maximum stress overall. Strain transition values led to significant anisotropic manifestation post-treatment, where circumferential tissues transitioned at higher strains compared to axial counterparts. Hysteresis values and energy loss decreased after enzymatic treatment, where hysteresis reduced by almost half of the untreated value. Anisotropic ratios exhibited axially led stiffness at low strains which transitioned to circumferentially led stiffness when subjected to higher strains. Viscoelastic stress relaxation was found to be greater in the circumferential direction for bronchial airway regions compared to axial counterparts.

**Conclusion:**

Targeted fiber treatment resulted in mechanical alterations across the loading range and interactions between elastin and collagen connective tissue networks was observed. Providing novel mechanical characterization of elastase and collagenase treated airways aids our understanding of individual and interconnected fiber roles, ultimately helping to establish a foundation for constructing constitutive models to represent various states and progressions of pulmonary disease.

## Background

Respiratory illnesses, including chronic obstructive pulmonary disease (COPD), account for several leading causes of death worldwide [1, 2, 3, 4]. These diseases are primarily characterized by compromised lung constituents due to remodeling, causing irreversible tissue damage and detrimental effects on lung mechanical properties [5, 6, 7, 8]. These alterations affect the primary stress bearing connective tissue network composed of collagen and elastin fibers, which are responsible for the structural integrity and elasticity of the lung, respectively [9, 10, 11].

One characteristic of pulmonary disease is the inability of lung tissue to recoil due to elastin and collagen fiber remodeling [12, 13, 14]. For example, emphysema and cystic fibrosis exhibit targeted elastin damage, activating the body’s physiological response to compensate through the addition of collagen fibers [15, 16, 17]. Paradoxically, fiber deposition strengthens the vulnerable areas, but leads to tissue stiffening, and decreases lung compliance [18, 19, 20]. This remodeling of the connective tissue network induces heterogeneity that exacerbates ventilator injuries, atelectasis, and other mechanical changes such as increased resistance and heightened strains in the lungs [21, 22, 23, 24, 25]. A study has also observed lung tissue breakage under normal breathing forces when elastin was damaged, making it easier for the lung to be overdistended without a healthy connective tissue network [26].

Exploring the lung tissue’s microstructural properties and simulating disease damage can enable an improved understanding of these pathological conditions. Enzymatic treatment of collagenase and elastase is a common means of exploring the role of elastin and collagen fibers in diseased states, and past studies have investigated the effect of this treatment on the aorta, cartilage, tendon, and spine [27, 28, 29, 30, 31]. Previous lung-focused experiments have extensively analyzed parenchymal strips in animals [32, 33, 34] and human lungs [18, 35], but the pulmonary airway tissues have not yet been mechanically analyzed and subjected to enzymatic treatment despite being the major site of obstruction and compromised lung function [36, 37, 38]. Examinations of healthy airways are scarce, let alone investigations of damaged states [39]; and the few studies which do exist of healthy tissue, explore the trachea, absent of the analysis of the distal airway tree [39, 40]. Additionally, past considerations have nearly exclusively been focused on uniaxial measurements [39, 41], and healthy airway tissue properties have only recently been analyzed biaxially to better represent physiological loading conditions [42].

To this aim, this current study compares healthy airway tissues to their corresponding biochemically-induced diseased states by conducting biaxial tensile tests on the various regions of the airway tree to examine the heterogenous and anisotropic mechanical behavior for the first time. By employing fiber-specific enzymatic degradation treatments, collagenase and elastase, we comprehensively characterize airway tissue mechanical properties via elasticity, anisotropy, and energetics. This study of collagen and elastin airway mechanics provides insights regarding the specialized role of individual connective tissue fibers and network interactions. The comparable quantitative insights obtained from this study provide a key steppingstone towards creating structurally motivated constitutive models representative of various states and progressions of pulmonary diseases [43, 44].

## Methods

### Sample preparation

**Fig. 1:**
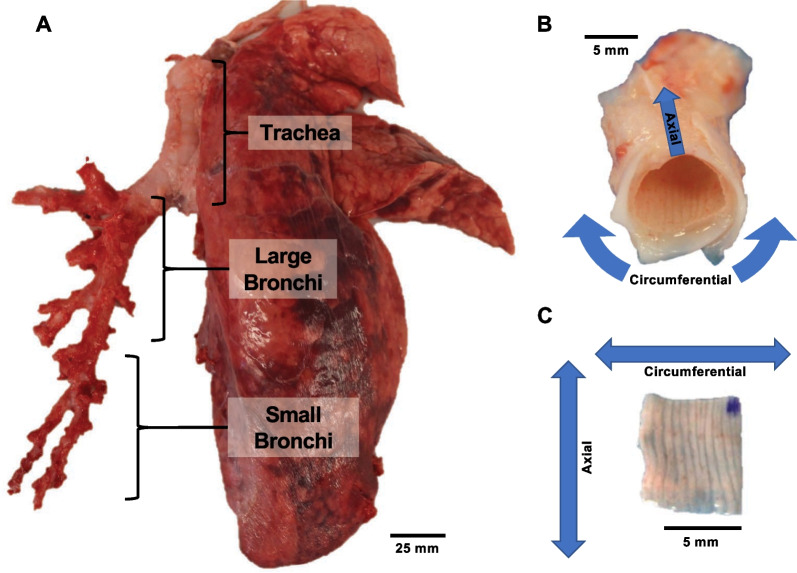
**A** The whole porcine lung with the left lung’s parenchyma removed reveals the main bronchial network. Three main airway regions are shown: the trachea large bronchi, and small bronchi. **B** Bronchial section with soft tissue attached to the cartilage, and **C** the isolated soft tissue specimen with denoted circumferential and axial orientation which was subjected to biaxial testing

Porcine airway tissue samples were collected from six lungs of healthy pigs weighing 200–250lbs and 6–8 months of age (IACUC approval not required). The stiff airway cartilage was carefully separated to isolate the soft connective inner tissue, which was later subjected to mechanical testing [45]. The extracted airway specimens were cut with an effective square testing size of 5.2 ± 0.7 mm. The tissues were categorized into three regions based on their extra- or intra-parenchymal location and airway inner diameters [39]: trachea (18.6 ± 2.6 mm), large bronchi (8.5 ± 1.7 mm), and small bronchi (4.9 ± 0.9 mm) (Fig. 1A). Six specimens were collected from each trachea, and three samples each were collected for the small and large bronchi per lung lobe. The left and right lobes were found to have no significant differences; hence, lobes were combined to obtain six samples per bronchial region. Collectively, 108 total samples were tested across all pigs.

The circumferential and axial orientation of each sample was indicated with a corner mark (Fig. 1B, C) to examine anisotropy in addition to regional heterogeneity. Samples were stored at – 20 ℃, which has observed no remarkable change in mechanical properties for similar tissues [46, 47]. Prior to experimentation, all tissues were transferred and thawed 24 h in advance in a 4 ℃ refrigerator. Samples were further thawed at room temperature and submerged in 1X phosphate-buffered saline (PBS) before biaxial testing to maintain the tissue’s properties and minimize any degradation from freezing [48].

### Enzymatic treatment

For direct comparison with their untreated counterpart, once the samples were tested, each lung region’s samples were equally divided into a collagenase and elastase group and treated (Worthington Biochemical Corp.). The enzymatic solutions were prepared using deionized water with a concentration of 96 units/ml for collagenase and 0.1 units/ml for elastase including 0.001 mg/ml Trypsin inhibitor [49]. Both solutions were mixed using a thermal shaker at 100RPM and 37 ℃. Enzymatic treatment involved a fully submerged airway sample post-biaxial testing in a 37 ℃, 1 ml bath of either the collagenase solution for 30 min, or elastase solution for 1 h [27].

### Mechanical testing

Displacement controlled biaxial mechanical testing of the porcine airway tissue samples were conducted using 1.5N load cells (CellScale Biomaterials Testing, Waterloo, Canada) with five metal rake tines to minimize initial tissue inhomogeneities from sample loading [50]. To ensure a consistent reference state among samples, load cells were zeroed prior to sample loading and an initial 5.2 mm square testing size was used. Each sample underwent five preconditioning cycles followed by a subsequent analyzed test cycle all at 1%/s to ensure reproducibility and observation of the tissue’s repeatable behavior [51, 52, 53]. Specimens were then subjected to the same preconditioning protocol at a faster 5%/s loading rate preceding a 5 min hold to assess viscoelastic stress relaxation [40, 42, 54]. Preliminary tests sought to find a universal loading protocol suitable for all various tissue region samples; certain regions were observed to tear at 70% stain and thus, 60% equibiaxial strain was designated as the peak deformation load. Once affixed to the rakes, the samples were submerged in a 37℃ PBS bath throughout testing to mimic natural body temperature [52].

### Data analysis

**Fig. 2:**
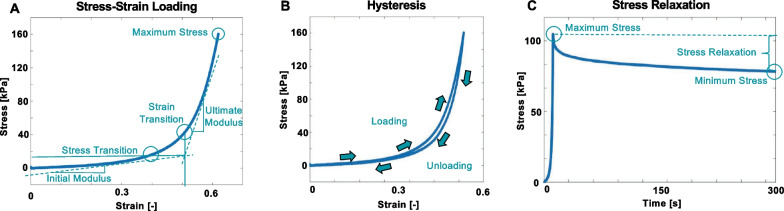
**A** A representative stress–strain loading profile of an airway tissue where the stress and strain transitions were calculated by determining the intersection of the initial and final modulus slopes from a bilinear fit of the low- and high-strain portions of the curve. Maximum stress was defined as the stress value at 60% strain with a strain rate of 1%/s. **B** A representative cyclic loading cycle utilized to measure tissue hysteresis and calculate energy loss. **C** A representative viscoelastic hold used to calculate the stress relaxation with a loading strain rate of 5%/s

Measures of interest extracted from the stress–strain loading curve were shown in Fig. 2A, where a transition between low and high strains is noted. MATLAB (MathWorks, Natick, Massachusetts, USA) was used to compute the bilinear initial modulus and ultimate modulus slopes as in prior studies where a linear fit with R^2^ > 0.90 was used [31, 55, 56]. The intersection of the bilinear fits determined the corresponding strain and stress transition values [42, 57]. Maximum stress was defined at 60% strain. Figure 2B depicted the calculation of hysteresis as the area between the loading and unloading curves [58] and energy loss was calculated by normalizing the hysteresis value by the area under the loading curve [59]. A representative viscoelastic tissue holding sequence in Fig. 2C illustrates tissue stress depletion over 300 s, where the difference between the maximum stress value and asymptotic stress value is normalized by the maximum stress value at the end of the hold to calculate the stress relaxation [40, 54, 60]. The anisotropic ratio was calculated as the ratio of the circumferential to the axial direction of each stiffness moduli (initial and ultimate), and defined as < 1 as anisotropic and axially leading, 1 as isotropic, and > 1 as anisotropic and circumferentially leading [61].

### Statistical analysis

Statistical analysis of the mechanical characteristics in the study were conducted using a 2-way paired ANOVA when comparing treated versus untreated groups, while an unpaired 2-way ANOVA was used to compare directional and regional statistical differences. A post-hoc Bonferroni adjustment was used for all tests with defined significance of *p < 0.05, **p < 0.01, ***p < 0.001, and ****p < 0.0001.

### Histological analysis

Representative samples from each lung region were collected to qualitatively observe untreated original structures compared to elastase and collagenase treated tissues. Samples were fixed in 10% neutral buffered formalin immediately after testing and submerged in 70% ethyl alcohol. Samples were sent to the University of California Irvine Experimental Tissue Resource Center to be embedded in wax paraffin blocks and cut into two slices collected onto glass slides. The samples were axially oriented to visualize the axially aligned connective tissue fibers [41]. Sections were stained with Masson’s Trichrome, exhibiting collagen fibers in grey and elastin fibers in blue. Slides were scanned (Ventana DP200 ROCHE) and analyzed using the QuPath (University of Edinburgh, Scotland) software [62]. The scans were analyzed with MATLAB using global thresholding to visualize the elastin and collagen fibers separately, producing high contrast binary images of each fiber network [63].

## Results

**Fig. 3:**
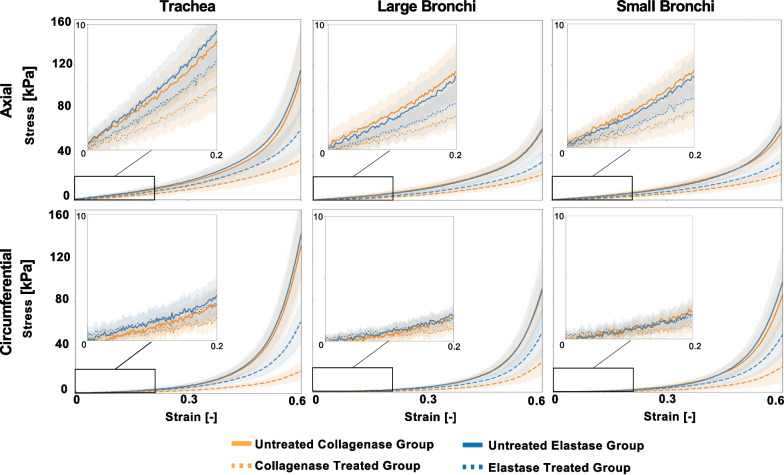
**A** Average ± standard deviation stress–strain loading profiles of untreated, collagenase treated, and elastase treated porcine airway specimens for all lung regions shown in both axial and circumferential stretch directions. The untreated tracheal region exhibited the highest stresses while the small and large bronchi shared similar stress–strain profiles. In the large deformation region (strains > 0.3), enzymatic treatments tended to result in decreased stress values for all lung regions and directions compared to untreated counterparts

Porcine airway tissue stress–strain loading profiles of the trachea, large bronchi, and small bronchi in both circumferential and axial stretch directions for untreated and treated elastase and collagenase samples were compared (Fig. 3). Enzymatic treatment tended to reduce stress values across the same displacement range, however the elastase treated circumferentially stretched groups were not observably different than the untreated samples at small deformations (< 0.3 strain). When initially loaded, axial stresses were greater than circumferential counterparts. In contrast, at 60% strain, the average circumferential stresses surpassed axial stresses for all regional counterparts except for the collagenase treated trachea.

**Fig. 4:**
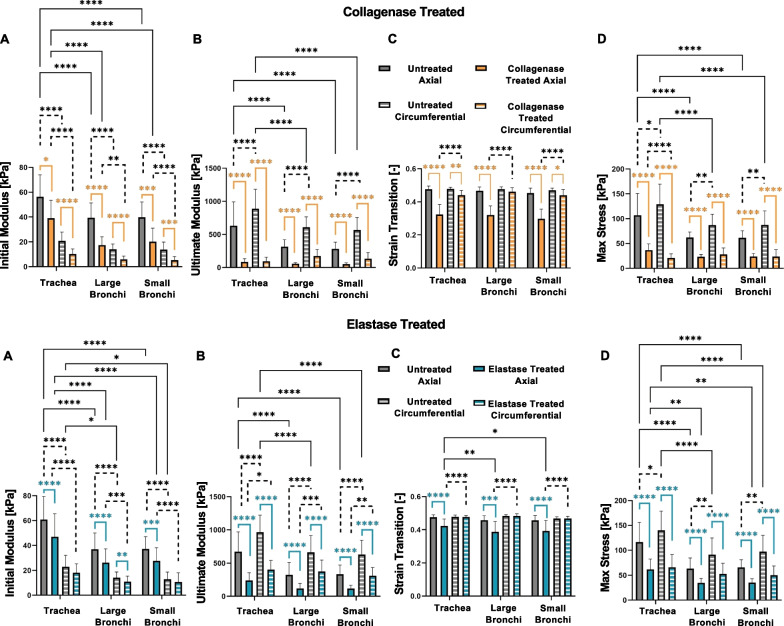
Average ± standard deviation of the initial (**A**) and final modulus (**B**), strain transition (**C**), and maximum stress (**D**) of each enzyme treatment, lung region, and stretch orientation. Significances are colored for treatment, dotted for directional, and solid for regional differences. Enzyme treatment generally resulted in decreased tissue stiffness lower stresses, and decreased strain transitions

Mechanical properties of the airways were analyzed by comparing the initial modulus, ultimate modulus, strain transition, and maximum stress values for treatment, directional, and regional dependencies in Fig. 4. Within each treated or untreated groups, the initial modulus (Fig. 4A) was significantly greater axially compared to circumferentially for all tested regions, which aligns with sheep airway distension during deep inspiration [64]. The initial modulus decreased significantly with collagenase treatment for all regions and directions. Similarly, elastase treatment was observed to significantly reduce initial modulus in the axial direction for all three regions and was also significantly reduced in the circumferential direction for the large bronchi. Comparisons between regions revealed the axially stretched trachea exhibited significantly greater initial modulus compared to the bronchial airways, which was maintained after enzymatic treatment.

The ultimate modulus (Fig. 4B) in the untreated groups were significantly greater than the collagenase and elastase treated counterparts, similar to the initial modulus. However, unlike the initial modulus where its highest values were in the axial direction, the ultimate modulus was significantly greater in the circumferential orientation after elastase treatment and trended greater after collagenase treatment. Regionally, the untreated trachea had greater ultimate modulus values than either the large or small bronchi; post-treatment however, the tracheal ultimate modulus declined greatly, even noted to become more compliant circumferentially after collagenase treatment compared to the bronchial airways.

The bilinear behavior of each airway sample exhibited a transition in tissue stiffness between 30–50% strain. Strain transition values (Fig. 4C) were significantly higher for untreated axial samples compared to treated collagenase and elastase counterparts. Similarly, significantly reduced strain transitions were also seen in the circumferential direction for collagenase treated trachea and small bronchi regions, with a reduced trend for the large bronchi. Enzymatically treated circumferential strain transition values were significantly greater than the axial direction, whereas the untreated samples had no directional differences. The tracheal samples in the elastase treated axial orientation had significantly higher strain transition values than the large and small bronchi.

Maximum stresses decreased 50% or more after enzymatic treatment (Fig. 4D, Table 1). Untreated circumferential samples experienced significantly higher maximum stresses than their axial counterparts, where post-enzymatic treatment resulted in generally similar directional behaviors. Maximum stress regional differences were observed in the untreated groups as well as within the axial elastase treated samples. The tracheal region had significantly higher maximum stresses overall which resembled that of the bronchial airways post-treatment except for the fact that the circumferential maximum stress was lower than the small and large bronchi post-collagenase treatment, which was not seen on other regions or in post-elastase treatment.

**Table 1:**
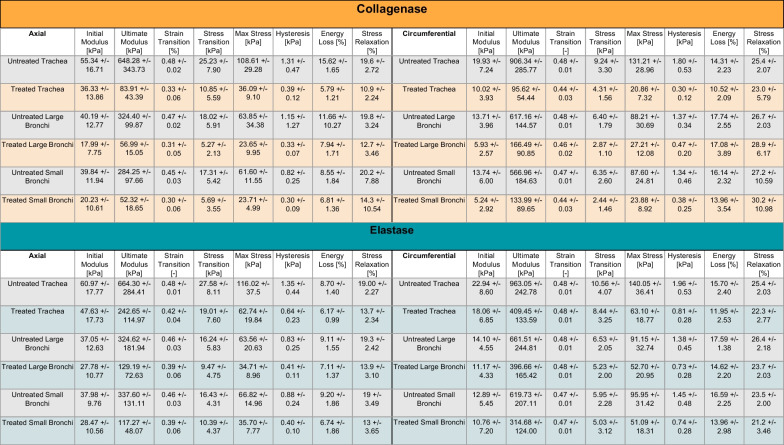
Treated and untreated specimen groups’ loading curve, energetics, and stress relaxation measurements

Average ± standard deviation values of initial and final moduli, strain and stress transition, maximum stress, hysteresis, energy loss, and stress relaxation of each treatment, lung region, and tissue orientation

**Fig. 5:**
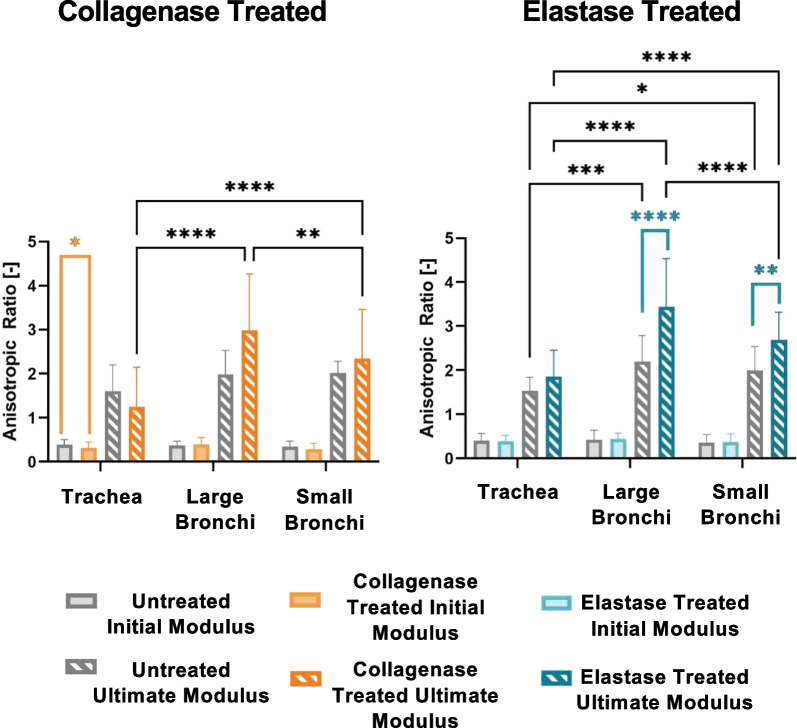
Average ± standard deviation of the anisotropic ratio for the initial and final moduli for each lung region as determined by the circumferential divided by the axial direction. The initial modulus anisotropic ratio was less than one, exhibiting greater axial values, while the ultimate modulus anisotropic ratio was greater than one indicative of circumferentially leading anisotropy

Figure 5 documents the anisotropic ratios of the initial and ultimate moduli, defined as the ratio between the circumferential and axial response. The initial modulus exhibits axially leading anisotropy in all specimens at low strains, which transitioned to a circumferentially led stretch at higher strains for the ultimate modulus (Table 2). Collagenase treated trachea resulted in a significantly lower anisotropic initial modulus ratio than its untreated counterpart, a trend that was also seen in collagenase treated small bronchi. The ultimate modulus anisotropic ratios of the elastase groups were significantly greater post-treatment for the two bronchial regions, and also notable in the trachea. Regional significances were only found in the ultimate modulus anisotropic ratios where the collagenase and elastase treated large bronchi had the greatest anisotropic ratio while trachea had the lowest ratio overall.

**Table 2:**
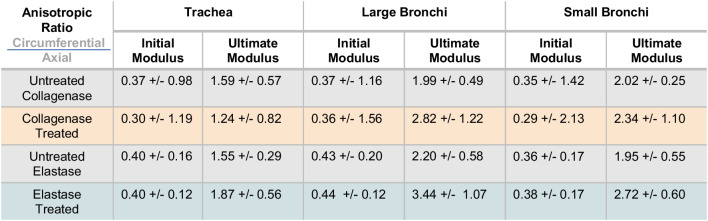
Treated and untreated groups’ anisotropic ratio

Average ± standard deviation of the anisotropic ratio values for the initial and ultimate moduli exhibited axially leading behavior at lower strains and circumferentially leading tissue behavior at higher strains, respectively

**Fig. 6:**
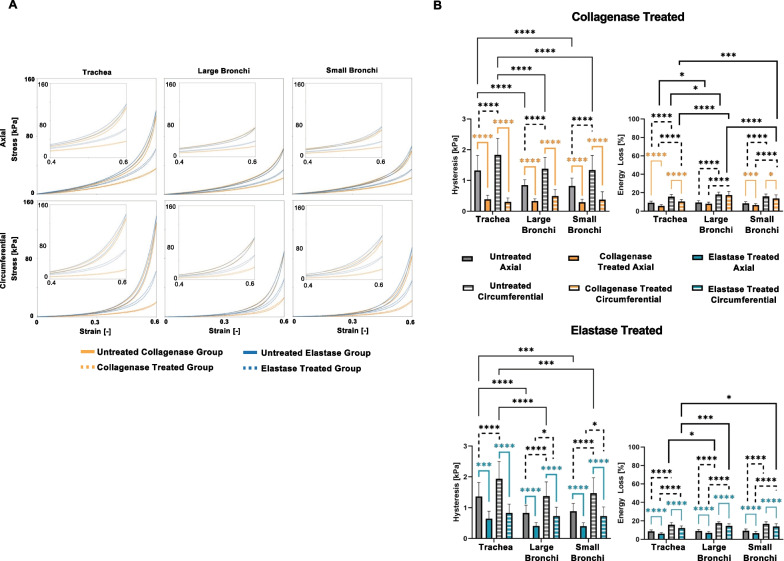
**A** The average cyclic loading and unloading stress–strain response of various lung regions, orientations and pre- and post-treatment responses and **B** average ± standard deviations of the hysteresis and energy loss between treatment, region, and orientation is shown. Hysteresis decreased after enzyme treatments in both orientations. Decreased energy loss was also evident post-treatment for all directions and regions and was lowest for the axial orientation

Airway tissue energetics were explored for each treatment group, region, and orientation in Fig. 6. The untreated trachea exhibited significantly greater hysteresis overall. Each untreated region exhibited significantly larger hysteresis values in the circumferential direction compared to the axial, which was also true for the elastase treated bronchial regions. Enzyme treatment resulted in a significant reduction in hysteresis, by nearly half, after collagenase and elastase treatment (Table 1).

The percent energy loss for the untreated groups was significantly greater than the treated groups for all except the collagenase treated large bronchi. Significantly lower tracheal energy loss was found in comparison to the large bronchi in all untreated circumferential groups and both collagenase treated stretch directions. Directionally, treated and untreated circumferential samples experienced significantly greater energy loss than axial counterparts across all regions. Post-treatment, circumferentially stretched samples observed significantly higher energy loss in the bronchial airway sections compared to the trachea, with the greatest energy loss in the large bronchi.

**Fig. 7:**
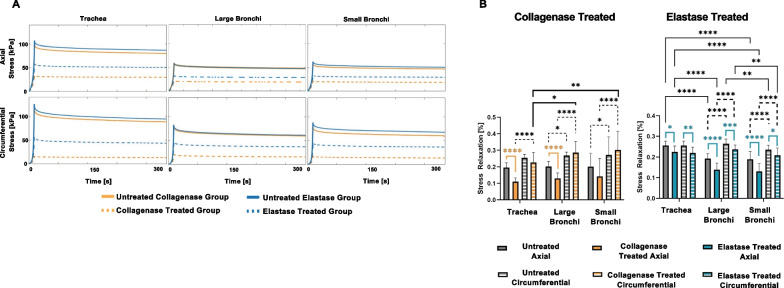
**A** Stress relaxation measured during a five-minute hold to examine viscoelastic tissue behavior. **B** Average ± standard deviation of the stress relaxation percentages based on treatment, region, and orientation. Elastase treatment resulted in significant decreases in stress relaxation across all samples, while collagenase treatment did not exhibit such a uniform response. Circumferential stress relaxation was significantly higher than its axial counterpart in the bronchial regions regardless of treatment

Tissue viscoelasticity was explored via pre- and post-treatment tissue stress relaxation in Fig. 7. Peak stress values mirrored that of maximum stress values in Fig. 4 and Table 1, despite undergoing a faster strain rate of 5%/s. Interestingly, collagenase treatment resulted in similar axial stress values during the hold for all three regions. Elastase treatment specifically resulted in significantly reduced stress relaxation percentages across all sample parameters, similarly true for the axially stretched large bronchi and tracheal collagenase treatment groups. Circumferentially stretched bronchial regions of the collagenase group exhibited remarkably higher stress relaxation trends compared to the untreated samples. The bronchial airways showed significantly greater relaxation percentages in the circumferential orientation compared to the axial direction regardless of enzyme treatment. This directional behavior was also significant in the collagenase treated tracheal group. Both collagenase treated large and small bronchi regions expressed significantly larger stress relaxation than the trachea in the circumferential orientation. Treated and untreated large bronchi in the elastase group had significantly greater stress relaxation than the small bronchi in the circumferential stretch direction and significantly lower relaxation than the trachea in the axial direction. The tracheal stress relaxation was also significantly higher than the small bronchi in the axial direction pre- and post-elastase treatment.

**Fig. 8:**
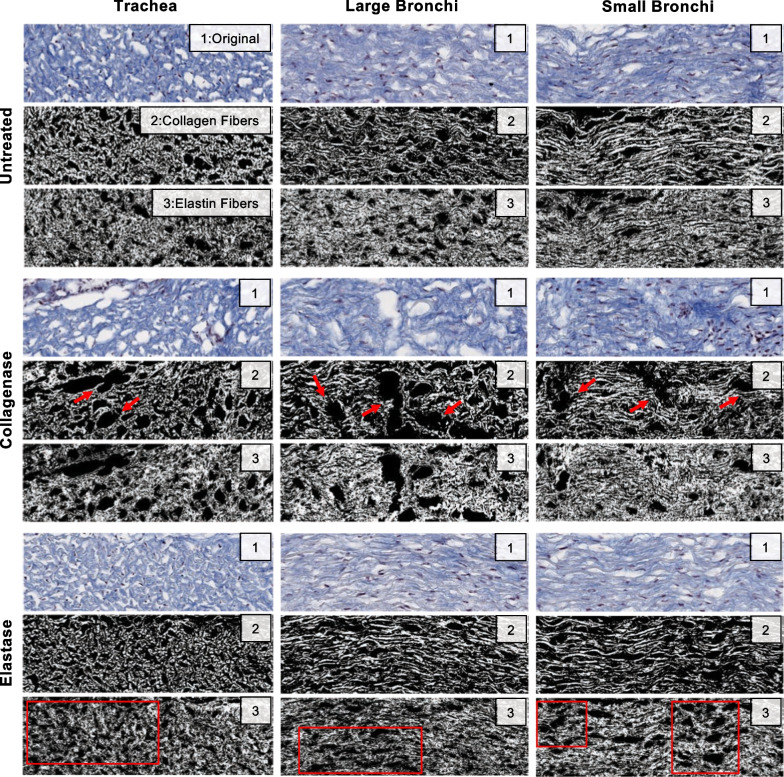
Representative histological images of the airway tissue samples across lung regions provided qualitative assessment of the effect of treatment. First row original scans are depicted in blue (denoted by 1: Original), and the second and third rows employed thresholding to enhance visualization and separate the collagen and elastin fibers into binary images (denoted by 2: Collagen Fibers and 2: Elastin Fibers). Red arrows indicate observed collagen fiber tears and fiber network gaps, and the red squares highlight sections of sparse elastin fiber content when contrasted to untreated counterparts

Qualitative visualization of enzymatic treatment and fiber degradation were considered in representative histological images (Fig. 8). The untreated collagen and elastin fibers were seen to exhibit a generally consistent fiber network, with uniform weave and density. The collagenase treatment resulted in severed collagen fibers, where a sparser network with sites of damage and irregular gaps were observed compared to the untreated counterparts of the trachea, large bronchi, and small bronchi regions. Elastase treated samples similarly demonstrated depletion of the elastin network with widespread voids compared to untreated counterparts.

## Discussion

This study explores the isolated and interconnected roles of the elastin and collagen fibers in the airways by examining pre- and post-enzyme treated biaxial mechanical properties for the first time. Resulting mechanical characteristics, energetics, and viscoelastic comparisons of healthy and disease-emulating tissue with respect to orientation and region, provides microstructural insights regarding pulmonary tissue damage. These finding improve our understanding of respiratory diseases characterized primarily by these structural changes of the elastin and collagen fiber networks.

### Extensibility and structural role of elastin

Elastin is the main contributor to the elastic recoil and extensibility of the lung, therefore, its degradation or alterations in the elastic fiber assembly are reported to cause hyper-distension, loss of elasticity, and increased lung compliance in emphysema and COPD patients [65, 66, 67]. Similarly, we observe the same phenomena of increased lung compliance marked by a significant reduction in initial and ultimate modulus for all elastase treated airway regions (Fig. 4A, B). A study of elastase treated parenchymal tissue strips report parallel findings to that of our airway studies and link the moduli reduction with the disruption in intact elastic fibers [33]. Our representative histological images of the airways also demonstrate such voids, as well as fragmentation in the elastin network (Fig. 8).

Morphologically healthy airway tissues exhibit a coarse elastin fiber network throughout the lung where the loading forces are distributed homogeneously [39, 68, 69]. We qualitatively observe a less dense elastin network post-treatment (Fig. 8), potentially affecting various regions of the airways to differing degrees and preventing homogeneous force dispersion. Regional strain transition differences emerge between the trachea and bronchi post-treatment (Fig. 4C), which were not observed prior to treatment in the control group; additionally, the post-elastase circumferential tissue group transitions to high stress conductance at smaller strains compared to pre-treatment. Furthermore, the strain transition exhibits significant anisotropy post-treatment, as well as consistent anisotropy at high strains in the ultimate modulus in accordance with a previous aorta study describing a greater degree of stiffness in the circumferential direction [70]. Elastase treatment also leads to significant regional heterogeneity between all regions for the anisotropic ultimate modulus ratio (Fig. 5), notably different between the large and small bronchi, which tended to behave similarly throughout the study.

### Morphology of collagen and its mechanical properties

Collagen fibers are characterized as the primary load bearing constituent in the lung, known to be crimped within the connective tissue network [71, 72, 73], as also seen in our histological images (Fig. 8). This configuration is important for lung function at the higher strain regime, as the wavy collagen fibers activate once fully elongated [22, 74], and protects the lung from overdistension [15, 23]. The bilinear stress–strain curve demonstrates the effect of collagen activation, resulting in increased stiffness at higher strains (Fig. 3). This has been previously observed in uniaxial and biaxial studies of the airway and lung tissue [33, 39, 42], while also exhibited in analogous bulk pressure–volume parenchymal tissue specimens subjected to saline [75] and air [76, 77, 78] inflation. As such, collagenase treatment has proven to disrupt collagen fiber load bearing properties and weaken tissue structural integrity, which ultimately leads to increased compliance, as also seen for the aorta and parenchyma [27, 33, 75]. Similarly, our results find significant reduction of both stiffness moduli and maximum stress values (Figs. 4A, B, D).

Collagenase treatment can also change collagen fiber crimp configuration; lower crimp angles and shorter fiber lengths have been reported to affect mechanical properties in diseased biological tissues [79, 80, 81, 82]. These changes in collagen fiber morphology results in premature fiber elongation, leading to the lower strain transition values we observe in our post-collagenase treated airway specimens (Fig. 4C), as also reported in tendon [83]. A combination of decreased fiber crimping and shortened axially-oriented fibers can additionally explain the loss of directional significances post-collagenase treatment within the ultimate modulus (Fig. 4B).

In addition to altered anisotropy, collagenase treatment also results in the loss of regional dependency, where previous differences between the trachea and bronchial airways in measurements of ultimate modulus, maximum stress, and hysteresis are no longer significant (Figs. 4B, and 6). Airway regional dependency in healthy tissues was previously linked to morphological differences between the proximal and distal airways, where collagen fibers were more crimped in the tracheal regions and cohesively straightened in the bronchial airways [41]: this is also evident in our histological images (Fig. 8). The structural degradation of collagen fibers potentially results in the homogenization and unification of proximal and distal airway mechanics.

### Interconnected connective tissue fiber roles

In addition to individual contribution, the cross-linking and interconnection between constituents of extracellular matrix (ECM), including collagen, elastin, and proteoglycans are responsible for the mechanical properties of the lung and undergo remodeling in pathological conditions [84, 85]. However, their contribution in various stages of stretching is debated: traditionally, collagen and elastin fibers are thought to act independently where elastin is engaged in the low strain regime and where collagen acts as a stop-length, preventing the lung from over distending [86, 87, 88]. As such, we expected to see significant material property changes in the collagenase treated groups only at the higher strain regime, and similarly to the elastase treated at low strains because of the individually targeted enzyme degradation [33]. Instead, we observe significant behavioral effects from the enzymatic treatment throughout the whole loading cycle, suggesting the mechanical connection and functional dependency of both fibers in the low and high strain regimes. This may be attributable to elastin fibers surrounding and interweaving with collagen fibers within the trachea and bronchial airways [39, 69]. In fact, past studies have observed alterations of the collagen fiber architecture as a result of the loss of elastin structural support when undergoing elastase treatment [35, 89, 90]. The unweaving of elastin from collagen can be detrimental to tissue integrity, weakening collagen’s load bearing threshold [75, 91], and thus accounting for the decreased maximum stresses and significantly lower hysteresis values.

We also find lower maximum stresses in the airways after enzymatic treatments, which was also seen in parenchymal strips [33]. This observation may seem trivial in collagenase treated specimens, given that collagen is far more stiff than elastin (two orders of magnitude) and its degradation would reduce stress conductance [23, 92]; however, the substantial decrease in maximum stress in elastase treated samples is rather unexpected, given elastin’s dominant role in the low strain regime and its minimal stiffness compared to collagen. This observation can be another indication of network involvement and interdependencies, where elastin and collagen fibers act as interconnected springs. Regardless of elastase or collagenase treatment, springs have been removed from the system, leading to lower stress for the same applied displacement [33]. Additionally, the anisotropic response of the lung when subjected to enzymatic treatment is studied for the first time in this biaxial investigation and we observe similarities between elastase and collagenase treatments: the enzymatic treatment strengthens airway directional preference.

Airway tissue energetics demonstrated greater efficiency after enzymatic treatment (Fig. 7). This reduction in hysteresis and energy loss can be attributed to understandings from scaffold investigations where collagen deposition resulted in increased energy loss [93].; thus, selective enzymatic removal of collagen with collagenase, or disruption in the elastin-collagen network with elastase, is likely to cause the reverse: improved energy efficiency. We also observe changes to airway regional differences, where post-collagenase treatment observed lower stress relaxation values in the trachea compared to the distal airways, while greater stress relaxation in the trachea was found in untreated and elastase-treated specimens. These mechanical modifications can be due to the distinct collagen fiber morphology in the trachea, as discussed earlier, and potentially cause for comparatively greater remodeling and network disruption due to collagenase treatment.

### Limitations

Lung diseases often express high collagen deposition as a physiological response to restrengthen the tissue after elastin degradation [15, 17]. Collagen deposition leads to stiffer airway tissue behaviors and lower lung compliance [15, 16, 18]. Our study is in line with these observations, where the implementation of collagenase treatment results in the converse and expected loss of elasticity; however, we are limited by the absence of a physiological response due to ex-vivo testing and cannot replicate collagen deposition. Caution should also be taken when comparing our results with studies conducted in-vivo, as boundary conditions differ and the gas–liquid interface does not exist ex-vivo [33]. Our upcoming studies focusing on in-vivo disease modeling using mice will replicate this collagen response and better examine physiological manifestation of abnormal lung function[94, 95]. Collagen, elastin, and similar smooth muscle quantification would also be a future avenue to explore to provide additional insights regarding the effects on tissue mechanical properties and intrinsic tone of the smooth muscle.

This study focused on displacement-controlled tissue testing whereas an enzymatic study on lung parenchyma considered force-controlled testing as well [33]. Such an examination can potentially offer additional insights regarding the structural response of the airways but was not implemented given the heterogenous response of the bronchial network: our focus on exploring various regions of the airways restricted the ability to force-match the tests since the trachea more readily conducts high forces without tearing, causing the small bronchi to incur tissue damage before reaching comparable forces with the trachea. Similarly, our study utilized samples from the main bronchus up to our defined small airway diameters. Due to the increased branching in smaller bronchi, we were unable to explore further distal airway regions which are conventionally termed small depending on the animal (< 4 mm diameter) [64].

## Conclusions

Lung diseases are often characterized by irreversible damage to connective tissue elastin and collagen fiber networks. Exploring the mechanical behavioral changes after enzymatic treatments provides us with an improved understanding of disease manifestation and the individual fiber roles for the first time for airway tissues. Similar to other biological organs, we find that elastase and collagenase treatment resulted in higher airway compliance. We also find that mechanical alterations occur throughout the loading profile regardless of enzyme treatment, indicating the interwoven and collaborative function of the connective tissue. These observations challenge the notion that elastin and collagen work individually, where elastin fibers are only responsible for the structural support and recoil at low strains, and collagen elongates and prevents overdistension of the lung at high strains. Collagen and elastin interdependency suggests that damage to one fiber indirectly impacts the performance mechanics of the other. These comprehensive measurements establish a foundation for constructing much needed structurally representative collagen and elastin degenerative constitutive models to improve our understanding of pulmonary disease progression.

## Data Availability

The data from this study are available upon reasonable request.

## Abbreviations

COPD: Chronic obstructive pulmonary disease
PBS: Phosphate-buffered saline
ECM: Extracellular matrix
